# Differences in the Thoracolumbar Fascia Between Low Back Pain Patients and Healthy Individuals: A Systematic Review with Meta-Analysis

**DOI:** 10.1186/s40798-026-01064-3

**Published:** 2026-08-17

**Authors:** Jan Wilke, Julius Debertshaeuser, Fabio Konrad

**Affiliations:** 1 Center of Sport Science, Department of Neuromotorics and Movement, University of Bayreuth, Universitätsstraße 30, 95444 Bayreuth, Germany; 2 Institute of Sport Sciences, Department of Movement Science, University of Klagenfurt, Universitätsstraße 63, 9020 Klagenfurt am Wörthersee, Austria

**Keywords:** Mechanical properties, LBP, Stiffness, Thickness

## Abstract

**Background:**

In view of new evidence revealing a mechanical and nociceptive role of the thoracolumbar fascia (TLF), it has been suggested to be involved in low back pain.

**Objective:**

To compare characteristics of the TLF in low back pain (LBP) patients and asymptomatic controls.

**Methods:**

Two independent investigators performed a systematic literature search using PubMed, Web of Science, Science Direct, Cochrane Library, and Google Scholar. Imaging studies (ultrasound/MRI) investigating the morphology and mechanics of the TLF in LBP patients and controls were included. We used robust variance estimation to pool (a) the standardized mean differences (SMD) between both groups and (b) the correlation between TLF characteristics and subjective pain ratings in LBP patients.

**Results:**

A total of 14 trials with moderate-to-high methodological quality (5.1 ± 1.2 points on the JBI critical appraisal checklist) were identified. Relative to asymptomatic controls, the LBP patients displayed a higher thickness (SMD: 0.64, 95% CI 0.41 to 0.88, *p* = < .001, 12 studies), stiffness (SMD: 0.82, 95% CI 0.34 to 1.31, *p* = .018, 3 studies), and echogenicity (SMD 0.43, 95% CI 0.16 to 0.70, *p* = .02, 3 studies) of the TLF. No effect was found for fascial layers sliding (SMD: − 0.05, 95% CI − 5.82 to 5.71, *p* = .92, 2 studies), although both studies of this comparison found significant (opposite) difference to controls. TLF thickening correlated with higher pain ratings.

**Conclusion:**

The TLF of patients with LBP is thickened, stiffened and more echogenic when compared to controls, while the role of shear mobility is unclear. As we additionally found associations of TLF characteristics with pain ratings, future research addressing the predictive value of these markers is warranted.

## Key Points

- Low back pain patients display increased stiffness, thickness, and echogenicity of the lumbar fascia.
- Shear mobility is altered, but evidence with regard to the direction of the effect is inconsistent.
- Properties of the lumbar fascia could represent a new factor in diagnostics and treatment of back pain, but prospective trials are needed to investigate their causal contribution.

## Introduction

Low back pain (LBP) is one of the most frequent musculoskeletal conditions. With a point prevalence of 18.3% and a one-month prevalence of 30.8% [1] it represents the leading cause of disability [2] and in 2050, it is projected to affect 843 million individuals [2]. Despite manifold efforts to unravel the pathomechanisms and to improve diagnostics, 9 in 10 cases are still labeled as nonspecific, meaning that no clearly identifiable cause can be determined [1]. So far, research on the somatic components of LBP has predominantly focused on muscles, bones, and spinal discs [3].

In recent years, deep fascia, the fibrous connective tissue surrounding skeletal muscle, gained significant attention as it has been demonstrated to play both a mechanical and a sensory role within the locomotor system. Creating a body wide network of myofascial tissue continuity, it is known to transmit forces across joints and to display autonomous capacities for stiffness regulation. With regard to the lower back, simulations of El Bojairami and Driscoll [4] found the thoracolumbar fascia (TLF) to represent the primary contributor to static spinal stability, even before muscle contraction and intraabdominal pressure generation. Regarding its sensory function, histologic analyses revealed a dense innervation with free nerve endings, which is threefold higher than in skeletal muscle [5] and positive staining for substance P and the calcitonin gene-related peptide suggests a nociceptive function [6]. Interestingly, chemical and electrical stimulation of the TLF in humans evoked stronger and longer lasting pain sensations than irritation of the erector spinae [7, 8].

In view of its possible implication in low back pain, imaging studies have increasingly targeted the TLF, examining a variety of morphological and mechanical parameters such as thickness, stiffness, echogenicity, and fascial layer shear mobility. However, there is no quantitative synthesis of the available evidence. This systematic review, therefore, aimed to compare the imaging-based characteristics of LBP patients and asymptomatic controls.

## Methods

A systematic review with meta-analysis, adhering to the PRISMA (Preferred Reporting Items for Systematic Reviews and Meta-Analyses) guidelines [9] was performed. It was prospectively registered in the Open Science Framework database (9F4ZR).

### Literature Search

Two independent examiners (FK, JD) screened the available literature using MEDLINE (PubMed), Web of Science, Cochrane Library, ScienceDirect, and Google Scholar from inception to April 2025. The search terms were similar but slightly modified to fit the requirements of the respective databases. As an example, we used the following string for PubMed: fascia AND (thickness OR stiffness OR elasticity OR "shear strain" OR "shear mobility" OR sliding OR gliding) AND ("back pain" OR CLBP OR LBP OR lumbalgia OR scoliosis OR lumbopelvic) AND (ultrasound OR elastography OR MRI OR "magnet resonance imaging"). For Google Scholar, the first 100 entries, ordered by relevance, were screened. Additionally, we checked the reference lists of all eligible articles identified by the database searches [10].

The inclusion criteria were: (1) cross-sectional study design, (2) enrolment of LBP patients and asymptomatic controls, (3) ultrasound or MRI measurements of TLF characteristics (i.e., thickness, stiffness, echogenicity, shear mobility), (4) publication in English or German in a peer-reviewed journal. Studies examining subjective outcomes or interventions (if baseline data were not separately reported) were excluded.

### Risk of Bias

The JBI critical appraisal checklist for analytical cross-sectional studies was applied to estimate the methodological quality of the included studies [11]. Disagreements between the two independent examiners (JD, FK) were resolved by discussion and if needed, a third investigator (JW) cast the decisive vote. To gauge the risk of a publication bias, we created and visually inspected contour-enhanced robust funnel plots (effect sizes against standard errors) considering the dependency of effect sizes. The criteria proposed by the GRADE working group [12] were used to classify the certainty about the evidence as very low, low, moderate, or high for each outcome. Certainty was downgraded by one level each in cases of risk of bias, imprecision, inconsistency, or indirectness. In contrast, large effect magnitudes led to an upgrade of one level.

### Data Extraction

Two independent investigators (JD, FK) performed the data extraction, obtaining sample size and participant characteristics, assessment methods (ultrasound/MRI) and TLF characteristics (thickness, stiffness, echogenicity, shear mobility) in patients and controls. If a study reported on several sub-groups (e.g. men and women) or had more than one outcome (e.g. thickness in two areas of the TLF), all data were obtained. From each study, we extracted the mean and standard deviation (SD) of the respective TLF characteristics for both LBP patients and controls, the mean difference and the SD of the difference between groups, and correlations between TLF characteristics and pain in the LBP group. In cases of missing data (i.e., SDs), the corresponding authors of the trials were contacted. If this was unsuccessful, values were (1) determined from figures by means of PlotDigitizer [13], (2) converted from *p* values/*t* values/standard errors, or (3) imputed following the recommendations of the Cochrane handbook.

### Data Synthesis and Statistics

Robust variance estimation (RVE) was applied to compute the standardized mean differences (SMD) and 95% confidence intervals (CI) between LBP patients and controls for the imaging-based outcome measures. RVE adjusts for dependency of multiple effect sizes by including a 'study' term as a random factor in the model [14]. Between-study heterogeneity was calculated by means of Tau^2^ while omega^2^ (ω^2^) was computed to express within-study variance [14]. Summary effect sizes (ES) were interpreted as small (SMD = 0.2 to 0.49), moderate (SMD = 0.50 to 0.79), or large (SMD ≥ 0.8, [15]). We also used RVE to examine the association of TLF characteristics and pain. To stabilize variance, Fisher’s z transformation was applied to correlation coefficients, and after meta-analytic pooling, resulting effect sizes and 95% CIs were back-transformed. Analyses were performed separately on Pearson's r and Spearman’s Rho. The software used was R (R Foundation for Statistical Computing, Vienna, Austria), with the packages metafor and robumeta [9] for meta-analysis, and dplyr and ggplot2 for funnel plots.

## Results

### Search Results

**Fig. 1:**
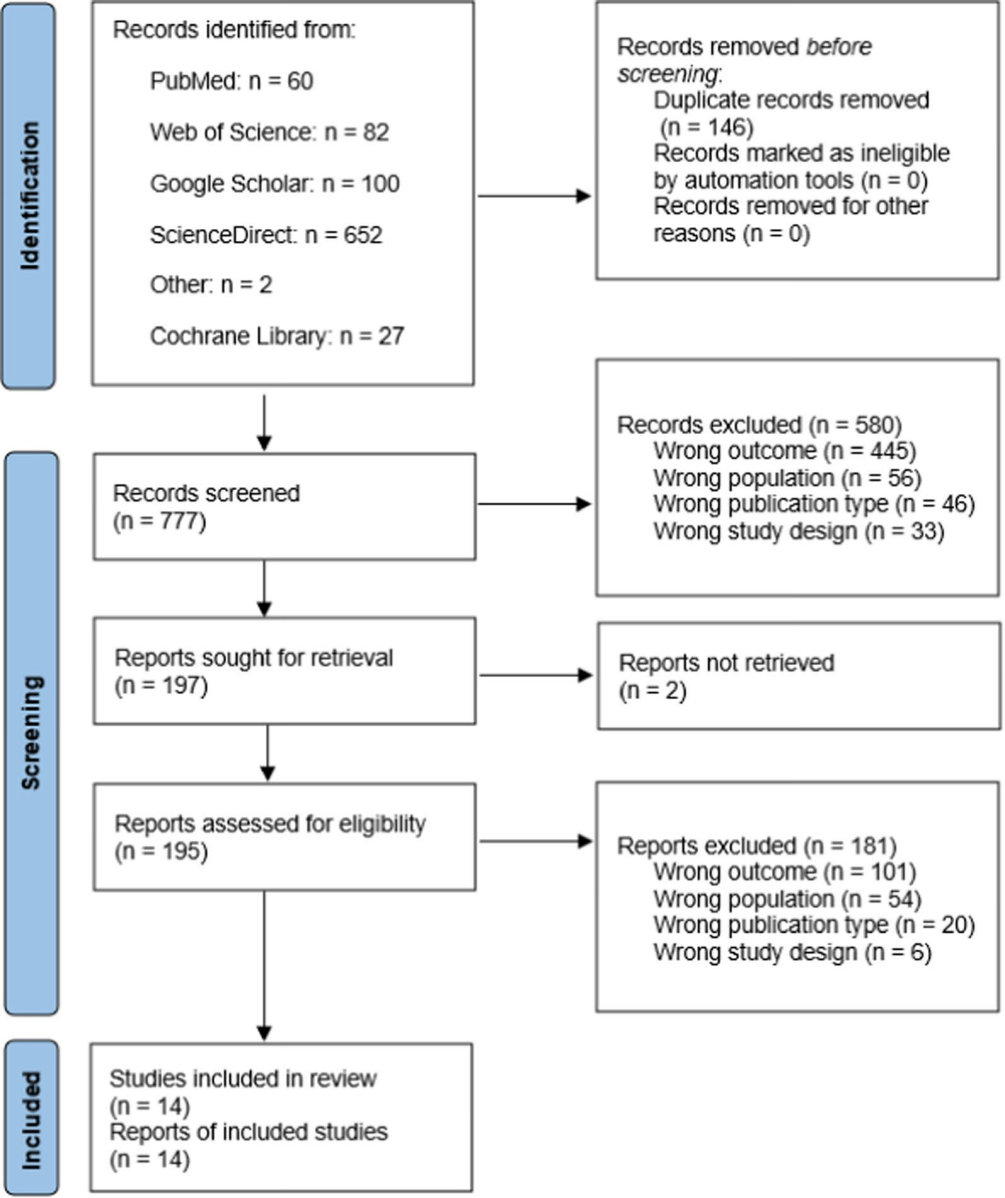
Flow of the literature search

A total of 923 articles were identified by the literature searches. After removal of duplicates and application of exclusion criteria, *n* = 14 studies were deemed eligible (Fig. 1).

### Characteristics of the Studies

The 14 studies cumulatively involved *n* = 1001 participants (451 males, 550 females, 24.93 ± 3.01 kg/m^2^, Table 1). All were performed on middle-aged adults except for one study recruiting adolescents [16]. Thirteen articles investigated both males and females, while Almazán-Polo et al. [17] examined only males. LBP pain was labelled as non-specific in all studies but one [16] which included patients with scoliosis. Six studies provided data on LBP duration and among these, the mean duration was 6.1 ± 5.1 years. Pain severity, assessed by means of different scales, was mostly moderate: five studies used the Oswestry Disability Scale (mean score: 27 ± 11), five applied a visual analogue scale (1–10, mean score: 5.7 ± 1.4) and four studies reported ratings on the McGill Pain Questionnaire (mean words chosen: 8.1 ± 2.4, mean pain score: 15.6 ± 6.1) The most investigated outcome was TLF thickness (12 studies), while stiffness (*n* = 3), sliding (*n* = 2), and echogenicity (*n* = 3) were less frequently captured.

**Table 1:** Study characteristics including anthropometrics of LBP and healthy subjects, measured outcomes, specifics of the utilized measurement system, as well as the pain persistence inclusion criteria

Study	Sample LBP	Sample healthy	Outcome	Measurement specifics	Pain persistence
Almazán-Polo et al. 2020 [17]	*n* = 15 male athletes 28 (10) yrs*, 181 ± 10 cm, 75 ± 10.9 kg, 23.85 (4.5) kg/m^2^*	*n* = 15 male athletes 24 (5) yrs*, 181 ± 7 cm, 78.95 ± 7.48 kg, 24.07 (1.83) kg/m^2^*	Thickness	Ultrasound Ecube i7, type L3_12T linear probe, 8–12 MHz	≥ one episode of recurrent pain per year in the past 2 years
Aslan et al. 2024 [18]	*n* = 30 (14 male, 16 female) 46.2 ± 10.6 yrs, 27.6 ± 3.9 kg/m^2^	*n* = 20 (10 male, 10 female) 41.9 ± 11.5 yrs, 26.6 ± 4.8 kg/m^2^	Thickness	Ultrasound Logice portable 5–12 MHz curvilinear transducer	≥ 3 months
Langevin et al. 2009 [19]	*n* = 60 (25 male, 35 female) 38.3 ± 13.3 yrs, 25.7 ± 0.6 kg/m^2^	*n* = 47 (20 male, 27 female) 39.3 ± 14.1 yrs, 25.9 ± 0.7 kg/m^2^	Thickness Echogenicity	Ultrasound Terason 3000 scanner, 10 MHz linear array transducer	History of recurrent LBP episodes or ≥ 1 year chronic LBP
Langevin et al. 2011 [20]	*n* = 71 (39 male, 32 female) 44.6 ± 1.8 yrs, 26 ± 0.5 kg/m^2^	*n* = 50 (24 male, 26 female) 41.8 ± 2.3 yrs, 26.1 ± 0.6 kg/m^2^	Thickness, Sliding, Echogenicity	Ultrasound Terason 3000 scanner, 10 MHz linear array transducer	Recurrent or chronic LBP for ≥ 1 year
Larivière et al. 2020 [21]	*n* = 35 (16 male, 19 female) *Male* 44.1 ± 13.5 yrs, 172 ± 6.2 cm, 76 ± 12.9 kg, 25.6 ± 3.8 kg/m^2^ *Female* 47.8 ± 13.9 yrs, 163.1 ± 5.8 cm, 71.4 ± 9.9 kg, 26.8 ± 3 kg/m^2^	*n* = 30 (15 male, 15 female) *Male* 39.3 ± 14.3 yrs, 178 ± 8.4 cm, 77.1 ± 10.7 kg, 24.4 ± 3.2 kg/m^2^ *Female* 39.8 ± 13.7 yrs, 164.2 ± 6.1 cm, 62.9 ± 10.9 kg, 23.3 ± 3.6 kg/m^2^	Thickness, echogenicity	Ultrasound Phillips HD11 1.0.6, 5–2 MHz curvilinear array transducer	≥ 4 weeks
Liu et al. 2024 [22]	*n* = 30 (19 male, 11 female) 27.40 ± 4.57 yrs, 22.96 ± 2.55 kg/m^2^	*n* = 32 (15 male, 17 female) 27.94 ± 4.94 yrs, 22.52 ± 2.26 kg/m^2^	Stiffness	Mindray, Eagus R9 Doppler ultrasound, L11-3U high-frequency linear array probe	≥ 3 months
Newell et al. 2023 [23]	*n* = 36 (16 male, 20 female) *Male* 50.56 ± 18.41 yrs, 177 ± 5.4 cm, 86.23 ± 15.63 kg, 27.71 ± 5.63 kg/m^2^ *Female* 45.50 ± 10.07 yrs, 164 ± 6.8 cm, 58.37 ± 7.65 kg, 26.39 ± 2.84 kg/m^2^	*n* = 33 (17 male, 16 female) *Male* 55.82 ± 15.11 yrs, 179 ± 0.7 cm, 84.18 ± 11.19 kg, 21.67 ± 2.25 kg/m^2^ *Female* 63.31 ± 13.7 yrs, 164 ± 8 cm, 61 ± 19.11 kg, 22.77 ± 7 kg/m^2^	Thickness	OsiriX MD (v12.0.3) MRI	Unknown
Perez et al. 2023 [24]	*n* = 23 (10 male, 13 female) 25.13 ± 10.04 yrs, 22.11 ± 2.84 kg/m^2^	*N* = 21 (12 male, 19 female) 22.94 ± 5.23 yrs, 22.30 ± 2 kg/m^2^	Thickness	Vinno E35 ultrasound device, linear probe 7–18 MHz	≥ 3 months
Pirri et al. 2023 [25]	*n* = 46 28.96 ± 10.54 yrs, 168.3 ± 4.83 cm, 66.22 ± 6.1 kg, 23.37 ± 5.22 kg/m^2^	*n* = 46 27.09 ± 12.38 yrs., 171.3 ± 6.76 cm, 70.60 ± 12.2 kg, 24.03 ± 6.1 kg/m^2^	Thickness	EDGE II, Sonosite high-resolution ultrasound device, 6–5 MHz	History of at least one episode per year of recurrent pain in the past two years
Tabesh et al. 2021 [26]	*n* = 60 (12 male, 48 female) 42.1 ± 11.3 yrs, 24.2 ± 4.2 kg/m^2^	*n* = 30 (6 male, 24 female) 41.2 ± 10.6 yrs, 24.7 ± 4.6 kg/m^2^	Thickness	Logiq E9 ultrasound machine device, linear probe ML6-15 MHz probe	Unknown (range: 3 months to 20 yrs)
Tamartash et al. 2022 [27]	*n* = 68 (33 male, 35 female) 40.2 ± 5.3 yrs, 171 ± 1.1 cm, 71.51 ± 12.09 kg, 24.86 ± 2.35 kg/m^2^	*n* = 63 (32 male, 31 female) 41.7 ± 4.9 yrs, 170 ± 0.8 cm, 71.74 ± 11.05 kg, 25.18 ± 1.53 kg/m^2^	Stiffness	SONON Ultrasound Imaging System, L14-5.38 linear array	≥ 12 weeks
Tomita et al. 2025 [28]	*n* = 33 (11 male, 21 female) 57.3 ± 9.3 yrs, 165.2 ± 8.6 cm, 85.3 ± 19.9 kg, 31.4 ± 7.1 kg/m^2^	*n* = 32 (10 male, 22 female) 51.4 ± 9.9 yrs, 163.7 ± 7 cm, 74.3 ± 16.4 kg, 27.8 ± 6 kg/m^2^	Thickness, sliding	Ultrasound Terason t3000 scanner, 12L5 linear array transducer	≥ 3 months
Yerli et al. 2024 [16]	*n* = 20 (4 male, 16 female) 17.1 ± 3.8 yrs, 166.8 ± 10.2 cm, 54.2 ± 11.2 kg, 19.3 ± 2.9 kg/m^2^	*n* = 20 (8 male, 12 female) 16.36 ± 4.69 yrs, 162.84 ± 13.17 cm, 55.94 ± 12.5 kg, 21.2 ± 3.9 kg/m^2^	Thickness	Unknown ultrasound system	> 3 months
Zandi et al. 2025 [29]	*n* = 15 (5 male, 10 female) 37.93 ± 6.68 yrs, 168.73 ± 8.83 cm, 74.6 ± 10.33 kg, 26.29 ± 3.94 kg/m^2^	*n* = 15 (5 male, 10 female) 32.93 ± 7.52 yrs, 166.4 ± 8.25 cm, 70.6 ± 9.48 kg, 25.49 ± 2.91 kg/m^2^	Thickness, stiffness	High-resolution ultrasound device, 2–10 MHz linear transducer	> 3 months

Anthropometric data are expressed as mean ± SD, * indicate median (interquartile range)

yrs :years, cm: centimeters, kg: kilogram, m: meter, MHz: megahertz, LBP: low back pain

### Methodological Quality and Risk of Bias

**Fig. 2:**
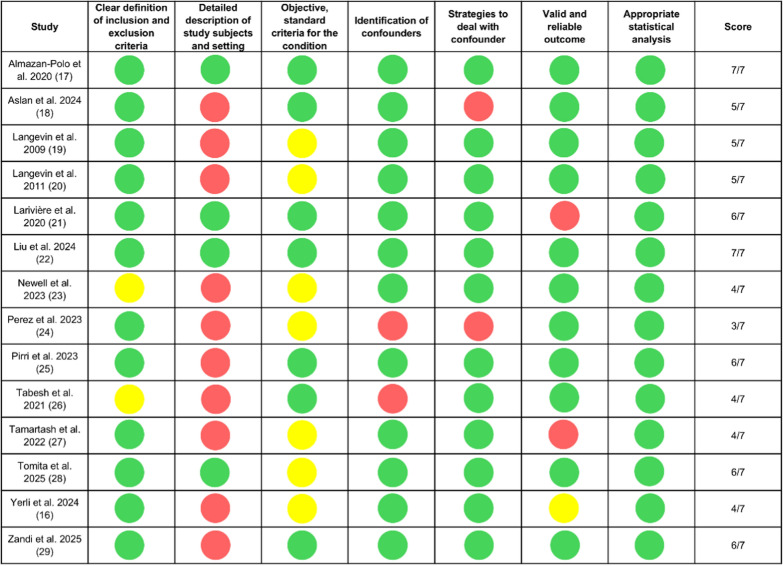
Ratings on the JBI scale for analytical cross-sectional studies. Colours indicate criterion fulfilment: green = full, yellow = partial, red = no

**Fig. 3:**
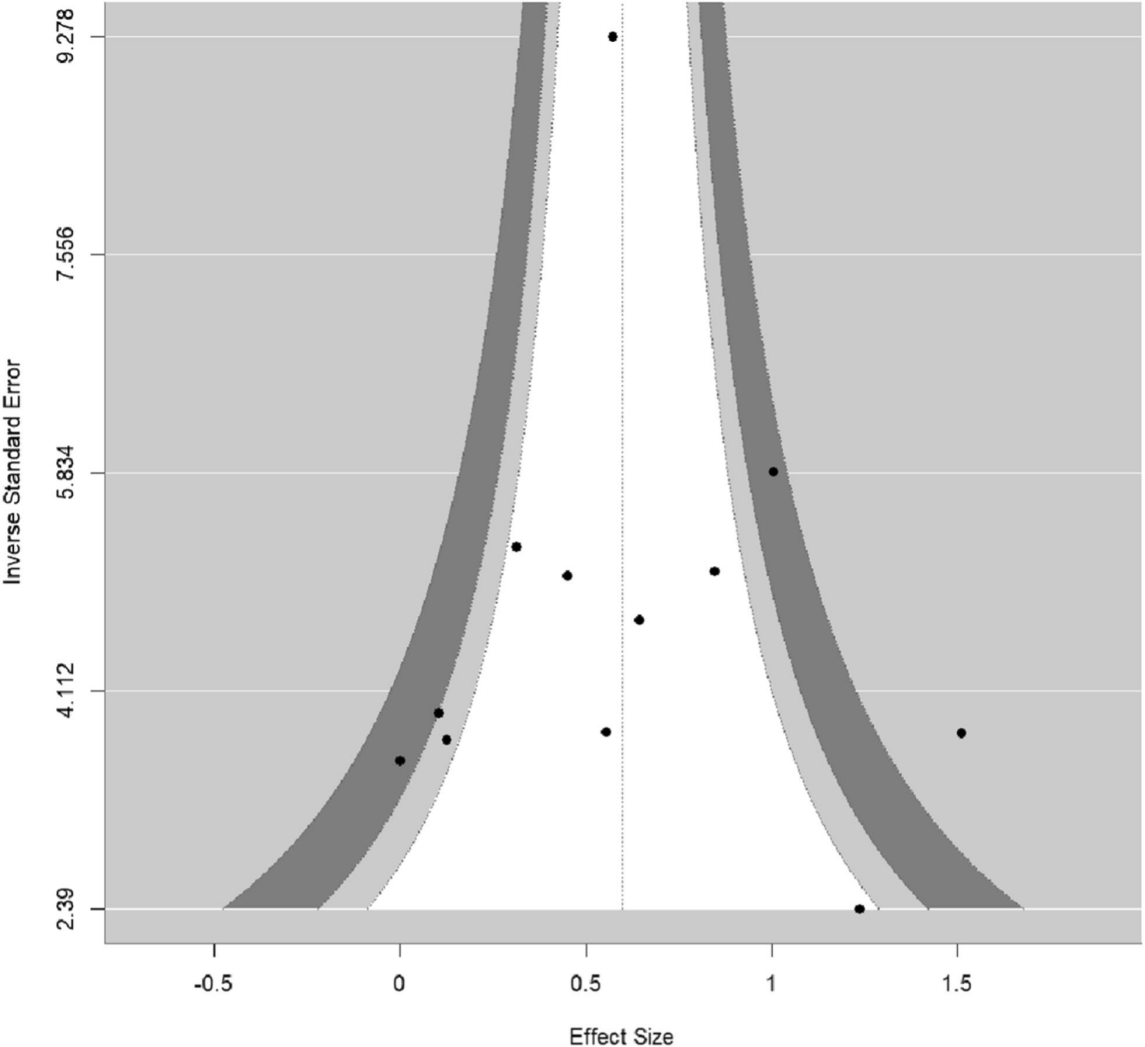
Contour-enhanced funnel plot for fascia thickness independent effect sizes

The mean JBI score was moderate to high with an average rating of 5.1 ± 1.2 out of 7 points (Fig. 2). Almost all studies provided clear definitions of inclusion and exclusion criteria, identified and appropriately handled confounders, used valid and reliable outcome measures, and applied appropriate statistical analyses. Main weaknesses included unclear reporting of sample characteristics (10 studies) and insufficient details on criteria for the measured condition (LBP, 7 studies). The contour-enhanced funnel plot for fascia thickness showed a symmetrical distribution of the aggregated effect sizes and no indication of a publication bias (Fig. 3). Regarding the other variables, the number of studies (< 10) was too low for inspection of plot asymmetry.

### TLF Differences Between Patients and Controls

**Fig. 4:**
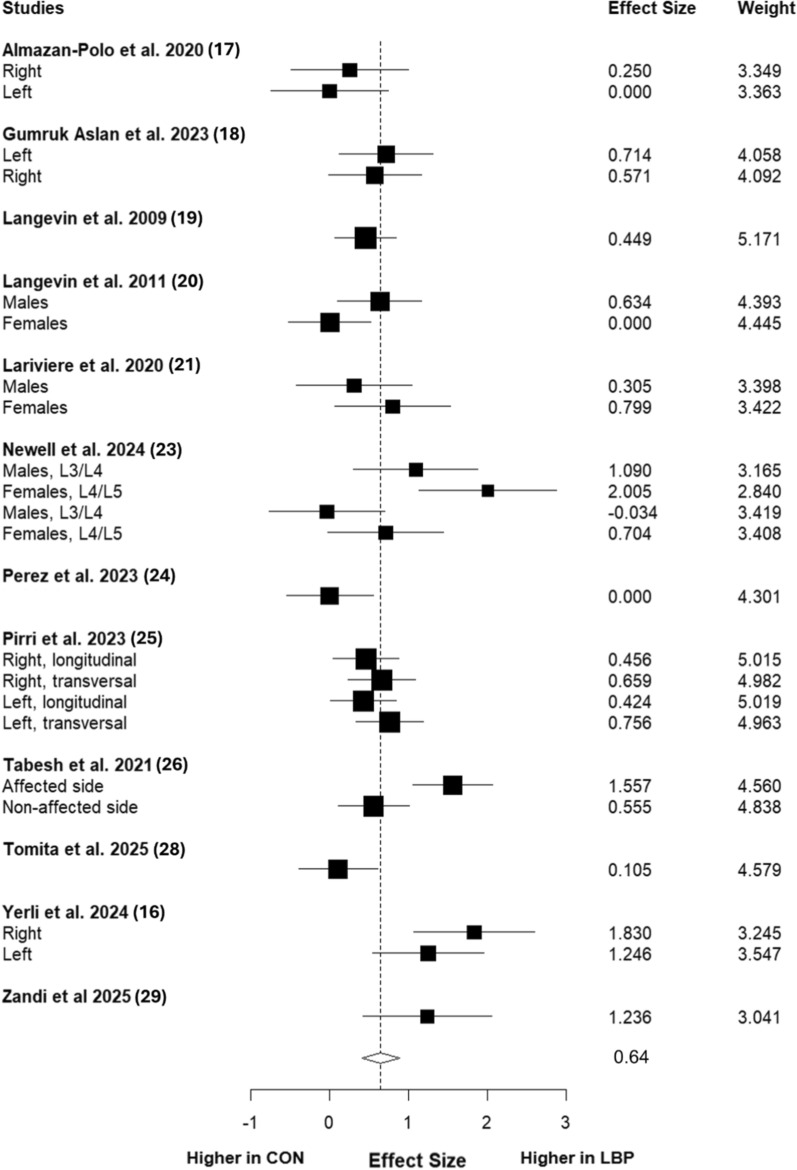
Forest plot displaying the TLF thickness difference in patients with low back pain (LBP) vs. asymptomatic controls (CON). TLF: thoracolumbar fascia, L3–L5 = 3rd to 5th lumbar vertebrae

**Fig. 5:**
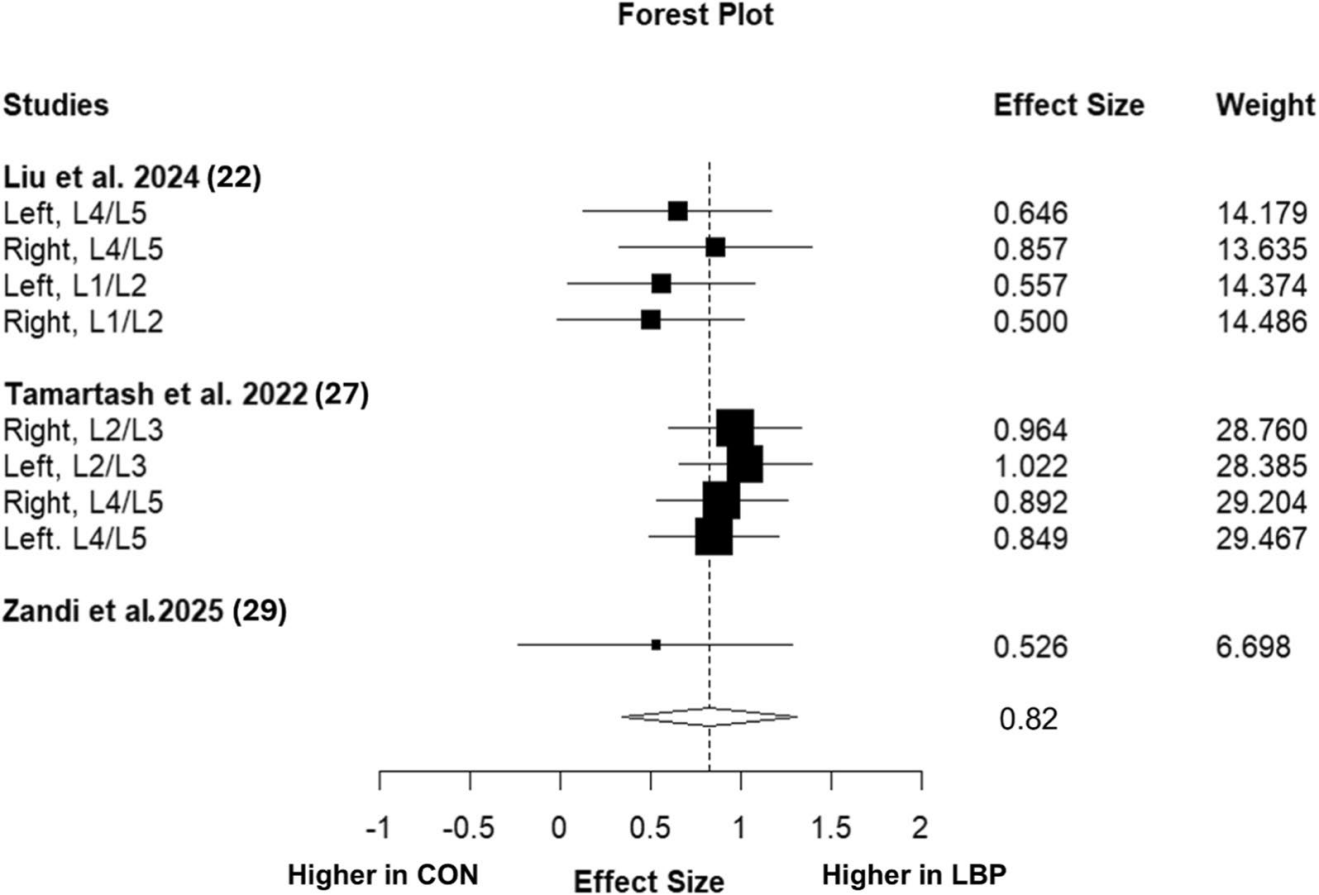
Forest plot demonstrating the TLF stiffness difference in patients with low back pain (LBP) vs. asymptomatic controls (CON). TLF: thoracolumbar fascia, L1–L5 =  1 st to 5th lumbar vertebrae

**Fig. 6:**
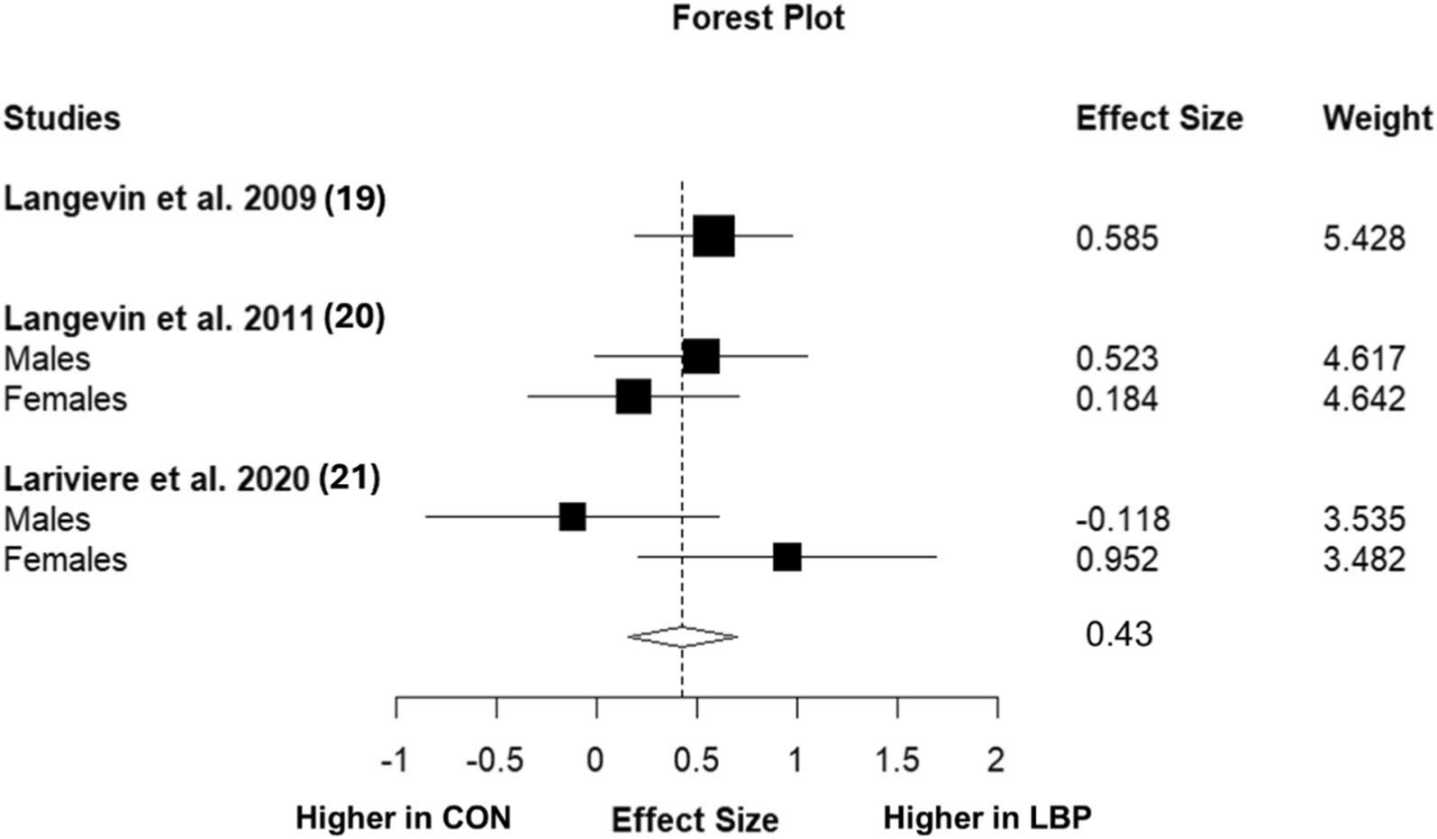
Forest plot illustrating the TLF echogenicity difference in patients with low back pain (LBP) vs. asymptomatic controls (CON). TLF: thoracolumbar fascia

**Fig. 7:**
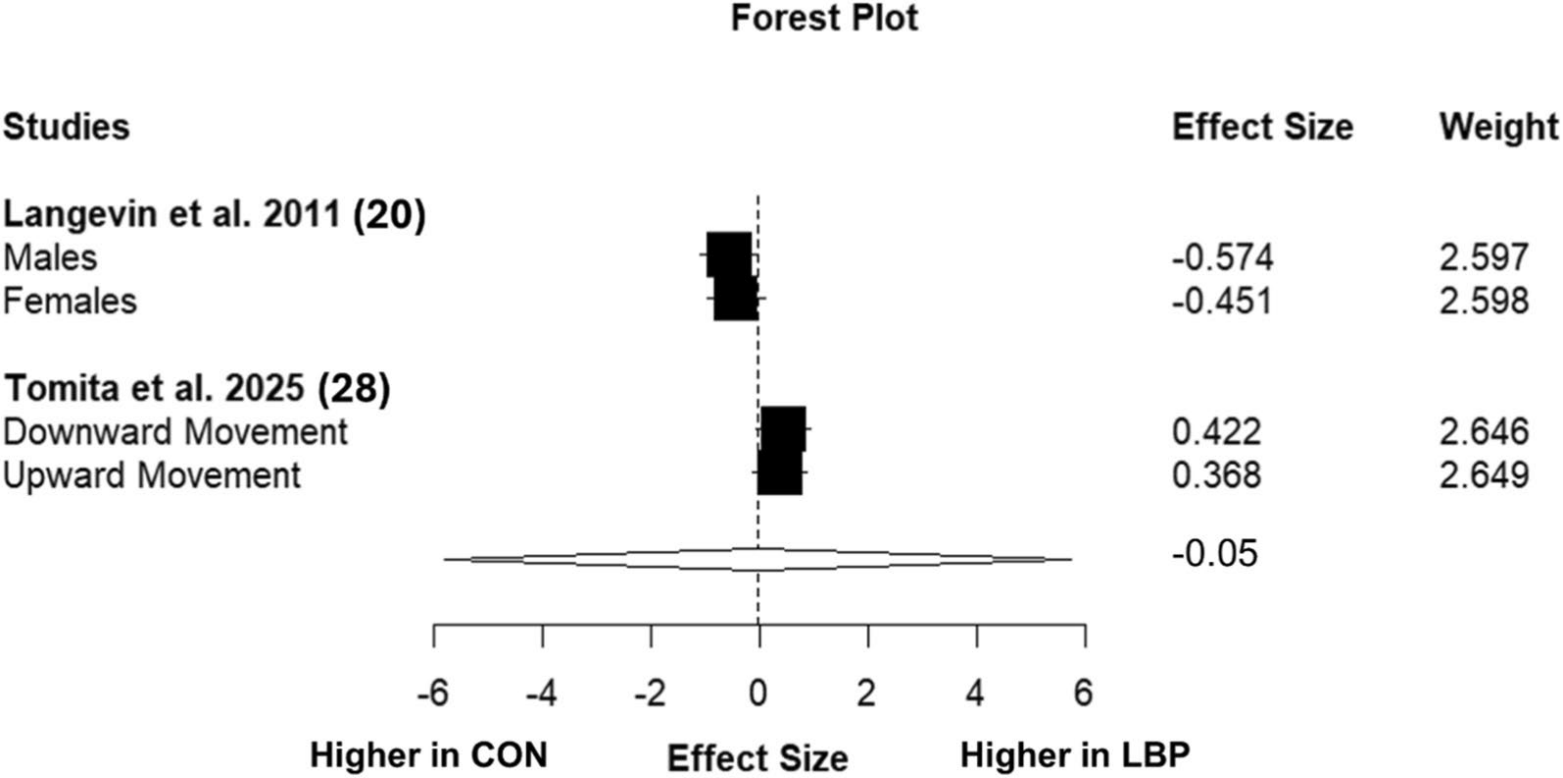
Forest plot showing the TLF sliding difference in patients with low back pain (LBP) vs. asymptomatic controls (CON). TLF: thoracolumbar fascia

Relative to controls, LBP patients displayed a higher thickness (SMD: 0.64, 95% CI 0.41 to 0.88, *p* =  < 0.001, 12 studies, 24 ES, **τ**^2^ = 0.09, **ω**^2^ = 0.06, Fig. 4), stiffness (SMD: 0.82, 95% CI 0.34 to 1.31, *p* = 0.018, 3 studies, 9 ES, **τ**^2^ = 0, **ω**^2^ = 0, Fig. 5), and echogenicity (SMD: 0.43, 95% CI 0.16 to 0.70, *p* = 0.02, 3 studies, 5 ES, **τ**^2^ = 0, **ω**^2^ = 0.14, Fig. 6) of the TLF. No difference was found for fascial layers sliding (SMD: − 0.05, 95% CI − 5.82 to 5.71, *p* = 0.92, 2 studies, 4 ES, **τ**^2^ = 0.31, **ω**^2^ = 0, Fig. 7), although both studies included in this comparison found significant (opposite) differences when compared to controls. The certainty about the evidence was high for thickness and stiffness and moderate for echogenicity (one downrank: lower bound of 95% CI included a trivial effect size).

### Association Between TLF Characteristics and LBP

**Fig. 8:**
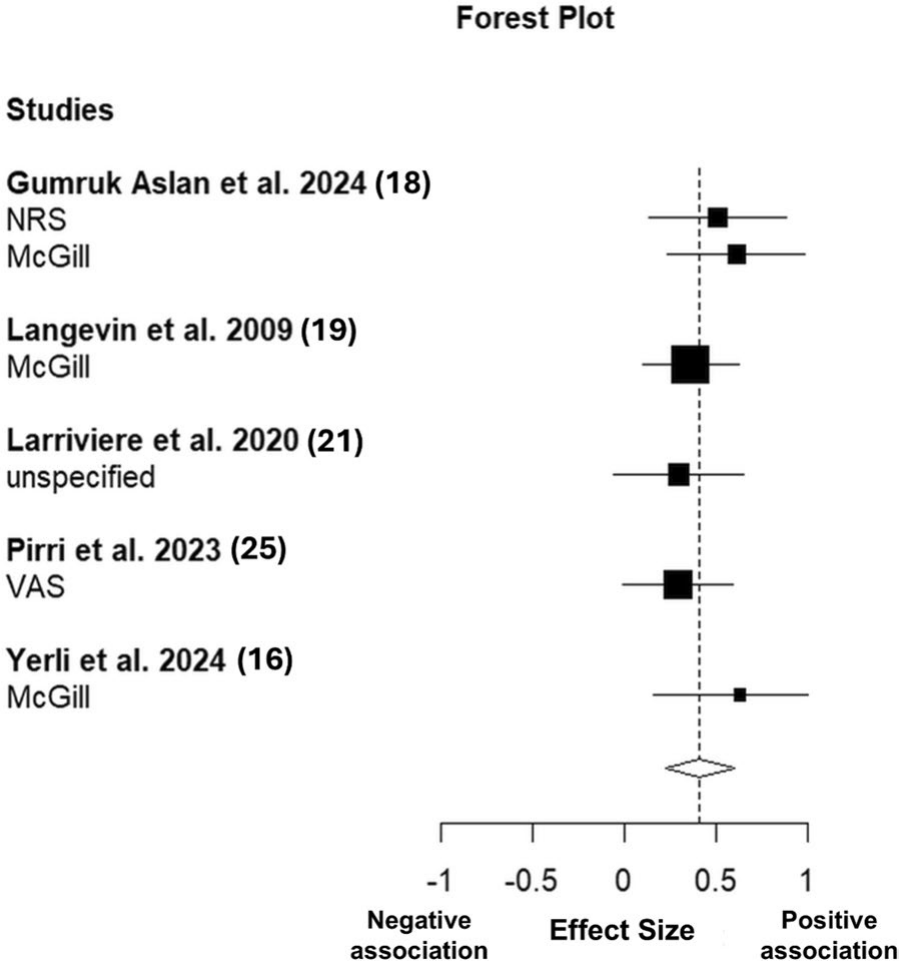
Forest plot presenting the association between TLF thickness and pain severity in patients with low back pain (LBP). TLF: thoracolumbar fascia, NRS: numeric rating scale (0–10 numerical pain rating), VAS: visual analogue scale (continuous pain rating), McGill: McGill Pain Questionnaire (multidimensional pain descriptor checklist)

Six studies [16, 18, 19, 21, 25, 30] examined the correlation between TLF thickness and pain. The article by Ushida et al. [30] reported Spearman’s rho and therefore, was not included in the quantitative summary. The authors found a large positive association between TLF thickness and pain ratings at the level L2/L3 (rho = 0.76) but no such effect at L4/L5 (0.08). Meta-analysis of the remaining five studies reporting Pearson coefficients revealed a moderate correlation of TLF thickness and pain (*r* = 0.39, 95% CI 0.23 to 0.54, *p* = 0.005, 5 studies, 6 ES, **τ**^2^ = 0, **ω**^2^ = 0, Fig. 8). The certainty about the evidence was high.

The relation between TLF stiffness and pain was investigated by two studies [22, 27]. The pooled estimate (*r* = 0.70, 95% CI 0.65 to 0.99, *p* = 0.10, 7 ES, **τ**^2^ = 0.01, **ω**^2^ = 0) marginally failed significance although both studies had consistent findings. The study by Tamartash et al. [27] provided data for the left and right body side and two levels (L2/L3 and L4/L5), respectively. A large positive association was identified for all four locations (*r* = 0.71 to 0.76). Similarly, Liu et al. [22] also examined both body sides and two levels (L1/L2 and L4/L5). While effect sizes were only moderate to large (*r* = 0.33 to 0.65), the result of a positive association between stiffness and pain was identical.

The two studies on shear strain [20, 28] both reported on association with pain. Yet, no meta-analysis was performed as they used different statistical approaches. In the study by Tomita et al. [28], linear mixed models regression revealed shear mobility as a predictor of the Brief Pain Inventory and Oswestry Disability Index scores. On the contrary, Langevin et al. [19] did not find significant associations of TLF shear mobility with pain intensity, pain level, or related disability. However, a negative correlation with pain duration was detected in males.

Regarding echogenicity, the only available study [19] found a weak association with pain duration (*r* = 0.30) but not with pain intensity.

## Discussion

This systematic review with meta-analysis is the first to compare the morphological and mechanical TLF characteristics of LBP patients with asymptomatic controls. We found that afflicted individuals display a higher thickness, stiffness, and echogenicity of the TLF, while results were ambivalent regarding fascial layer shear mobility. Of note, patients’ thickness correlated with the level of pain, suggesting a link between imaging-based findings and self-reported clinical symptoms.

The finding of an increased thickness of the TLF in patients vs. controls aligns with other pathologies as previous studies made similar observations in plantar fasciitis [31] and neck pain [32]. Fascial thickening has mainly been related to two processes. Fibrosis describes excessive production of collagen fibers while densification represents an increase in the loose connective tissue (LCT) between the fibrous layers [33]. Both fibrosis and densification may affect mobility and alter proprioceptive signaling and, in turn, lead to pain. Of note, they can be readily identified using ultrasound imaging, as the loose connective tissue and the hyaluronic nested within are hypoechoic, while collagen is hyperechoic [33, 34]. However, despite reporting the absolute stiffness of the TLF and echogenicity, the studies included in this review did not provide specific data on LCT thickness. Notwithstanding, interestingly, besides the enhanced thickness, we found a higher echogenicity in the TLF which may reflect fibrosis.

In addition to a higher thickness and echogenicity, patients had a stiffer TLF than controls. Again, this alteration in patients matches with other pathologies as individuals with plantar fasciosis also exhibit stiffening when compared to healthy controls [35]. While the higher stiffness may be related to both thixotropic changes and structural remodeling, mechanosensing allows cells to register changes in the properties of the extracellular matrix (ECM). Integrins, which are transmembrane proteins connecting the cytoskeleton to the ECM, function as transmitters of tissue strain [36]. Due to the direct structural linkage between ECM of the TLF and the embedded nociceptors, stiffening of the TLF may facilitate the discharge of the pain receptors. This fits with data from Finocchietti et al. [37] who found higher pressure pain sensitivity in muscles with harder vs. softer muscles and a meta-analysis of Vatovec and Voglar et al. [38] demonstrating higher stiffness of the trunk muscles in LBP patients compared to asymptomatic controls.

We did not find a difference between patients and controls regarding shear mobility. At first sight, this parameter may hence be deemed irrelevant for LBP. However, both studies included in the pooled comparison observed significant, but contrary alterations, in sum leading to a null result: Langevin et al. [20] detected less sliding of TLF layers in patients, whereas the recent investigation by Tomita et al. [28] reported opposite findings with higher shear mobility in patients. Firstly, the discrepancy may be due to different methodological approaches (measurement of displacement vs. shape change, slightly different range of interest placements). Researchers and clinicians interpreting the results and shear motion should therefore be aware that the set-up and parameters used in TLF shear mobility screening need to be clearly specified to facilitate interpretation. Secondly, it could also mean that both insufficient and excessive TLF layer motion are linked to LBP. Regarding the latter hypothesis, limited fascial sliding has been attributed to thixotropy of the hyaluronic acid located between the individual TLF and a higher viscosity may affect lumbar spine mobility and discharge of sensory receptors. Conversely, an excess in sliding may similarly affect proprioceptive and nociceptive input and compromise lower back stability. In sum, although the current evidence presents initial signs that TLF shear motion is altered in LBP patients, it is unclear if decreases or increases are pathognomonic.

Although evidence examining TLF screenings in patients with LBP is accumulating, there is still a need to better explain sources of heterogeneity (e.g., in fascial layer sliding). The use of ultrasound and MRI for examinations of fascia is relatively new; therefore, standardized protocols are essential to increase reliability of findings and facilitate comparisons between studies. For instance, although both studies examined shear mobility between the aponeurosis of the erector spinae and the TLF, Langevin and colleagues used the complete lumbar fascia while Tomita et al. selected the most superficial part only. Further relevant parameters to standardize include the orientation (longitudinal/transverse) and position of the ultrasound probe as scanning at different spinal levels may lead to significant variations in the examined parameters. Another pressing research deficit is a lack of studies allowing causal assumptions. The evidence presented here stems from cross-sectional data and therefore, it is unclear whether fascial alterations cause LBP or if they occur as a consequence of pain episodes. Prospective trials are needed to further delineate the cause-effect relationship between TLF morphology/mechanics and the occurrence of low back pain. Notwithstanding, our results have potential clinical implications. It is well-established that the vast majority of LBP cases are non-specific [39] and it would be plausible that a fraction of these could be related to alterations of the connective tissue. It has already been shown that the TLF is highly sensitive to nociceptive stimuli and the results presented here may provide the morphological substrate underlying LBP pathogenesis. While prospective trials are needed to confirm a cause-effect relationship, therapists treating patients may consider the TLF if other specific pathologies have been ruled out and traditional pain interventions have failed. This could include using interventions known to decrease stiffness (e.g., stretching or foam rolling) or exercise designed to improve multidirectional mobilization of the TLF.

## Conclusion

The morphology and mechanics of the TLF could represent a so far overlooked parameter of interest in patients with LBP. As demonstrated by the present meta-analysis, afflicted individuals display a variety of alterations such as thickening, stiffening and increased echogenicity. As we also found a correlation between TLF thickness and subjective pain ratings, future research examining the potential cause-effect relationship between imaging characteristics and LBP is warranted.

## Data Availability

All data are available upon request. Corresponding author can be contacted via email.

## Abbreviations

CI: Confidence interval
CON: Asymptomatic controls
ECM: Extracellular matrix
ES: Effect size
LCT: Loose connective tissue
LBP: Low back pain
L1–L5: 1St to 5th lumbar vertebrae
NRS: Numeric rating scale
PRISMA: Preferred Reporting Items for Systematic Reviews and Meta-Analyses
RVE: Robust variance estimation
SD: Standard deviation
SMD: Standardized mean difference
TLF: Thoracolumbar fascia
VAS: Visual analogue scale

## References

[1] Maher C, Underwood M, Buchbinder R. Non-specific low back pain. Lancet. 2017;389(10070):736–47. 10.1016/S0140-6736(16)30970-9. 27745712 10.1016/S0140-6736(16)30970-9

[2] Ferreira ML, De Luca K, Haile LM, Steinmetz JD, Culbreth GT, Cross M, et al. Global, regional, and national burden of low back pain, 1990–2020, its attributable risk factors, and projections to 2050: a systematic analysis of the Global Burden of Disease Study 2021. Lancet Rheumatol. 2023;5(6):e316–29. 10.1016/S2665-9913(23)00098-X. 37273833 10.1016/S2665-9913(23)00098-X PMC10234592

[3] Wilke J, Schleip R, Klingler W, Stecco C. The lumbodorsal fascia as a potential source of low back pain: a narrative review. BioMed Res Int. 2017;2017:1–6. 10.1155/2017/5349620. 10.1155/2017/5349620 PMC5444000 28584816

[4] Bojairami IE, Driscoll M. Coordination Between Trunk Muscles, Thoracolumbar Fascia, and Intra-Abdominal Pressure Toward Static Spine Stability. Spine. 2022;47(9):E423–31. 10.1097/BRS.0000000000004223. 34545044 10.1097/BRS.0000000000004223

[5] Barry CM, Kestell G, Gillan M, Haberberger RV, Gibbins IL. Sensory nerve fibers containing calcitonin gene-related peptide in gastrocnemius, latissimus dorsi and erector spinae muscles and thoracolumbar fascia in mice. Neuroscience. 2015;291:106–17. 10.1016/j.neuroscience.2015.01.062. 25681518 10.1016/j.neuroscience.2015.01.062

[6] Tesarz J, Hoheisel U, Wiedenhöfer B, Mense S. Sensory innervation of the thoracolumbar fascia in rats and humans. Neuroscience Oktober. 2011;194:302–8. 10.1016/j.neuroscience.2011.07.066. 10.1016/j.neuroscience.2011.07.066 21839150

[7] Schilder A, Hoheisel U, Magerl W, Benrath J, Klein T, Treede RD. Sensory findings after stimulation of the thoracolumbar fascia with hypertonic saline suggest its contribution to low back pain. Pain. 2014;155(2):222–31. 10.1016/j.pain.2013.09.025. 24076047 10.1016/j.pain.2013.09.025

[8] Schilder A, Magerl W, Hoheisel U, Klein T, Treede RD. Electrical high-frequency stimulation of the human thoracolumbar fascia evokes long-term potentiation-like pain amplification. Pain Oktober. 2016;157(10):2309–17. 10.1097/j.pain.0000000000000649. 10.1097/j.pain.0000000000000649 27322440

[9] Moher D, Liberati A, Tetzlaff J, Altman DG, for the PRISMA Group. Preferred reporting items for systematic reviews and meta-analyses: the PRISMA statement. BMJ. 2009;339:b2535. 10.1136/bmj.b2535. 19622551 10.1136/bmj.b2535 PMC2714657

[10] Horsley T, Dingwall O, Sampson M. Checking reference lists to find additional studies for systematic reviews. Cochrane Database Syst Rev. 2011. 10.1002/14651858.MR000026.pub2. 21833989 10.1002/14651858.MR000026.pub2 PMC7388740

[11] de Morton NA. The PEDro scale is a valid measure of the methodological quality of clinical trials: a demographic study. Aust J Physiother. 2009;55(2):129–33. 10.1016/s0004-9514(09)70043-1. 19463084 10.1016/s0004-9514(09)70043-1

[12] Guyatt GH, Oxman AD, Vist GE, Kunz R, Falck-Ytter Y, Alonso-Coello P, et al. GRADE: an emerging consensus on rating quality of evidence and strength of recommendations. BMJ. 2008;336(7650):924–6. 10.1136/bmj.39489.470347.AD. 18436948 10.1136/bmj.39489.470347.AD PMC2335261

[13] Aydin O, Yassikaya MY. Validity and reliability analysis of the PlotDigitizer software program for data extraction from single-case graphs. Perspect Behav Sci März. 2022;45(1):239–57. 10.1007/s40614-021-00284-0. 10.1007/s40614-021-00284-0 PMC8894524 35342869

[14] Hedges LV, Tipton E, Johnson MC. Robust variance estimation in meta-regression with dependent effect size estimates. Res Synth Methods Januar. 2010;1(1):39–65. 10.1002/jrsm.5. 10.1002/jrsm.5 26056092

[15] Faraone SV. Interpreting estimates of treatment effects: implications for managed care. P T Peer-Rev J Formul Manag. 2008;33(12):700–11. PMC2730804 19750051

[16] Yerli S, Yinanç SB, Yağcı G, Erbahçeci F, Özçakar L. Thoracolumbar fascia and chronic low back pain in idiopathic lumbar scoliosis: an ultrasonographic study. Eur Spine J Juni. 2024;33(6):2469–75. 10.1007/s00586-024-08266-x. 10.1007/s00586-024-08266-x 38653872

[17] Almazán-Polo J, López-López D, Romero-Morales C, Rodríguez-Sanz D, Becerro-de-Bengoa-Vallejo R, Losa-Iglesias M, et al. Quantitative ultrasound imaging differences in multifidus and thoracolumbar fasciae between athletes with and without chronic lumbopelvic pain: a case-control study. J Clin Med. 2020;9(8):2647. 10.3390/jcm9082647. 32823967 10.3390/jcm9082647 PMC7464501

[18] Gumruk Aslan S, Koylu Uyar S, Gurcay E. Potential role of thoracolumbar fascia in younger middle-aged patients with chronic low back pain. Int J Neurosci. 2024;134(11):1198–204. 10.1080/00207454.2023.2251671. 37606340 10.1080/00207454.2023.2251671

[19] Langevin HM, Stevens-Tuttle D, Fox JR, Badger GJ, Bouffard NA, Krag MH, et al. Ultrasound evidence of altered lumbar connective tissue structure in human subjects with chronic low back pain. BMC Musculoskelet Disord. 2009;10(1):151. 10.1186/1471-2474-10-151. 19958536 10.1186/1471-2474-10-151 PMC2796643

[20] Langevin HM, Fox JR, Koptiuch C, Badger GJ, Greenan- Naumann AC, Bouffard NA, et al. Reduced thoracolumbar fascia shear strain in human chronic low back pain. BMC Musculoskelet Disord. 2011;12(1):203. 10.1186/1471-2474-12-203. 21929806 10.1186/1471-2474-12-203 PMC3189915

[21] Larivière C, Preuss R, Gagnon DH, Mecheri H, Henry SM. Structural remodelling of the lumbar multifidus, thoracolumbar fascia and lateral abdominal wall perimuscular connective tissues: A cross-sectional and comparative ultrasound study. J Bodyw Mov Ther Oktober. 2020;24(4):293–302. 10.1016/j.jbmt.2020.07.009. 10.1016/j.jbmt.2020.07.009 33218526

[22] Liu K, Zhao T, Zhang Y, Chen L, Zhang H, Xu X, et al. Shear wave elastography based analysis of changes in fascial and muscle stiffness in patients with chronic non-specific low back pain. Front Bioeng Biotechnol. 2024;12:1476396. 10.3389/fbioe.2024.1476396. 39619623 10.3389/fbioe.2024.1476396 PMC11604440

[23] Newell E, Chorney H, Tiegs-Heiden CA, Benson JC, Ouellet J, Driscoll M. Augmentation of musculoskeletal soft tissue morphology within low back pain patients may suggest the presence of physiological stress shielding: An in vivo study. J Biomech Januar. 2024;162:111894. 10.1016/j.jbiomech.2023.111894. 10.1016/j.jbiomech.2023.111894 38070295

[24] Perez AM, Fernández-Carnero S, Sicilia-Gomez-de-Parada C, Cuenca-Zaldívar N, Naranjo-Cinto F, Pecos-Martín D, et al. Diaphragmatic activation correlated with lumbar multifidus muscles and thoracolumbar fascia by B-mode and M-mode ultrasonography in subjects with and without non-specific low back pain: a pilot study. Medicina (Mex). 2023;59(2):315. 10.3390/medicina59020315. 10.3390/medicina59020315 PMC9967570 36837516

[25] Pirri C, Pirri N, Guidolin D, Macchi V, Porzionato A, De Caro R, et al. Ultrasound imaging of thoracolumbar fascia thickness: chronic non-specific lower back pain versus healthy subjects; a sign of a “frozen back”? Diagnostics. 2023;13(8):1436. 10.3390/diagnostics13081436. 37189537 10.3390/diagnostics13081436 PMC10137552

[26] Tabesh OA, Ghossan R, Zebouni SH, Faddoul R, Revel M, Fayad F. Thoracolumbar fascia enthesopathy as a cause of low back pain: a retrospective and follow-up study. Fortune J Rheumatol. 2021. 10.26502/fjr.26880027.

[27] Tamartash H, Bahrpeyma F, Mokhtari DM. Ultrasound evidence of altered lumbar fascia in patients with low back pain. Clin Anat Januar. 2023;36(1):36–41. 10.1002/ca.23964. 10.1002/ca.23964 36199243

[28] Tomita N, Roy-Cardinal MH, Chayer B, Daher S, Attiya A, Boulanger A, et al. Thoracolumbar fascia ultrasound shear strain differs between low back pain and asymptomatic individuals: expanding the evidence. Insights Imaging. 2025;16(1):18. 10.1186/s13244-024-01895-2. 39812963 10.1186/s13244-024-01895-2 PMC11735703

[29] Zandi S, Mokhtarinia HR, Mosallanezhad Z, Mobin HK, Arab AM, Azadi F, et al. Reliability of shear wave elastography for cervical and lumbar fascia stiffness and thickness in healthy and chronic neck and low back pain subjects. J Bodyw Mov Ther. 2025;42:558–66. 10.1016/j.jbmt.2025.01.021. 40325722 10.1016/j.jbmt.2025.01.021

[30] Ushida K, Akeda K, Momosaki R, Yokochi A, Shimada T, Ito T, et al. Intermittent pain in patients with chronic low back pain is associated with abnormalities in muscles and fascia. Int J Rehabil Res. 2022;45(1):33–8. 10.1097/MRR.0000000000000507. 34860730 10.1097/MRR.0000000000000507

[31] Wearing SC, Smeathers JE, Sullivan PM, Yates B, Urry SR, Dubois P. Plantar fasciitis: are pain and fascial thickness associated with arch shape and loading? Phys Ther. 2007;87(8):1002–8. 10.2522/ptj.20060136. 17553919 10.2522/ptj.20060136

[32] Stecco A, Meneghini A, Stern R, Stecco C, Imamura M. Ultrasonography in myofascial neck pain: randomized clinical trial for diagnosis and follow-up. Surg Radiol Anat. 2014;36(3):243–53. 10.1007/s00276-013-1185-2. 23975091 10.1007/s00276-013-1185-2

[33] Pavan PG, Stecco A, Stern R, Stecco C. Painful connections: densification versus fibrosis of fascia. Curr Pain Headache Rep. 2014;18(8):441. 10.1007/s11916-014-0441-4. 25063495 10.1007/s11916-014-0441-4

[34] Pillen S, Tak RO, Zwarts MJ, Lammens MMY, Verrijp KN, Arts IMP, et al. Skeletal muscle ultrasound: correlation between fibrous tissue and echo intensity. Ultrasound Med Biol. 2009;35(3):443–6. 10.1016/j.ultrasmedbio.2008.09.016. 19081667 10.1016/j.ultrasmedbio.2008.09.016

[35] Thanwisate T, Siriwanarangsun P, Piyaselakul S, Tharmviboonsri T, Chuckpaiwong B. Plantar fascia thickness and stiffness in healthy individuals vs patients with plantar fasciitis. Foot Ankle Int. 2024;45(11):1270–8. 10.1177/10711007241274765. 39257080 10.1177/10711007241274765

[36] Selig M, Lauer JC, Hart ML, Rolauffs B. Mechanotransduction and stiffness-sensing: mechanisms and opportunities to control multiple molecular aspects of cell phenotype as a design cornerstone of cell-instructive biomaterials for articular cartilage repair. Int J Mol Sci. 2020;21(15):5399. 10.3390/ijms21155399. 32751354 10.3390/ijms21155399 PMC7432012

[37] Finocchietti S, Mørch CD, Arendt-Nielsen L, Graven-Nielsen T. Effects of adipose thickness and muscle hardness on pressure pain sensitivity: correction. Clin J Pain Oktober. 2011;27(8):735–45. 10.1097/AJP.0b013e31820c5353. 10.1097/AJP.0b013e31820c5353 21415721

[38] Vatovec R, Voglar M. Changes of trunk muscle stiffness in individuals with low back pain: a systematic review with meta-analysis. BMC Musculoskelet Disord. 2024;25(1):155. 10.1186/s12891-024-07241-3. 38373986 10.1186/s12891-024-07241-3 PMC10875766

[39] Balagué F, Mannion AF, Pellisé F, Cedraschi C. Non-specific low back pain. Lancet. 2012;379(9814):482–91. 10.1016/S0140-6736(11)60610-7. 21982256 10.1016/S0140-6736(11)60610-7

